# Endoscopic jejunal flap suturing for the treatment of refractory marginal ulcers—a case series

**DOI:** 10.1007/s00464-025-11831-0

**Published:** 2025-07-07

**Authors:** Tali Bar-On, Relly Reicher, Nathan Aviv Cohen, Shai Eldar, Danit Dayan, Adam Abu-Abeid, Sigal Fishman, Mati Shnell

**Affiliations:** 1 Internal Medicine T, Tel-Aviv Sourasky Medical Center, 6423906 Tel Aviv, Israel; 2 Bariatric Endoscopy Unit, Department of Gastroenterology and Liver Disease, Tel Aviv Sourasky Medical Center, Tel Aviv, Israel; 3 Bariatric Center, Division of General Surgery, Tel Aviv Sourasky Medical Center, 6423906 Tel Aviv, Israel; 4 Faculty of Medical and Health Sciences, Tel Aviv University, 69978 Tel Aviv, Israel

**Keywords:** Marginal ulcer, Endoscopic surgery, Bariatric surgery complications, RYGB, OAGB

## Abstract

**Background:**

Gastro-Jejunal anastomotic ulcer is a late complication of bariatric surgery, typically responsive to high-dose proton pump inhibitors (PPI). However, persistent ulcers may require surgery. Endoscopic suturing of a jejunal flap has recently shown promise. This study evaluates its implementation in our practice.

**Methods:**

We retrospectively analyzed 11 patients with refractory marginal ulcers (≥ 10mm) unresponsive to at least two months of high-dose PPI. In cases with a narrow anastomosis, a self-expandable metallic stent was added. Clinical success was defined as ulcer healing on endoscopy at 8 weeks, while failure included persistent ulcer, perforation, or need for surgery.

**Results:**

Seven patients had undergone Roux-en-Y gastric bypass (RYGB) and four had one-anastomosis gastric bypass (OAGB). Ulcers were diagnosed on average 33 months post surgery. All patients were H. pylori-negative and denied alcohol/NSAID use; four were active smokers. Patients had been on chronic PPI therapy for an average of 25 months. Jejunal flap suturing was performed in 10 patients, with three also receiving a stent. One patient underwent stent placement alone due to stricture to relief obstructive symptoms. The technical success rate was 90.9%, but clinical success was 45%, with four patients requiring surgery. Notably, all OAGB patients had endoscopic failure, while 5 of 7 RYGB patients had ulcer healing.

**Conclusion:**

Jejunal flap suturing showed moderate success in treating marginal ulcers, with a good safety profile. Larger studies are needed to corroborate these findings, especially in OAGB patients.

**Graphical abstract:**

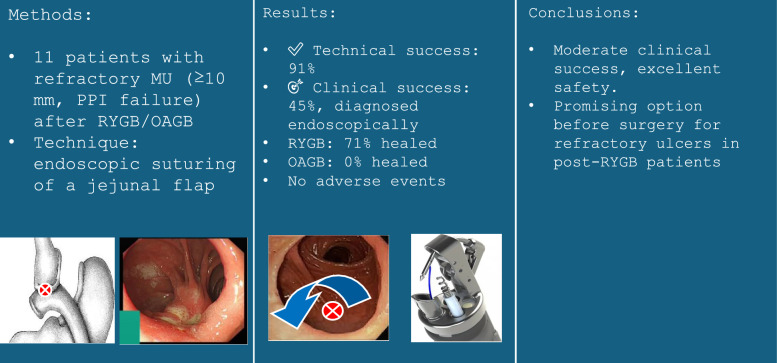

The development of a marginal ulcer is a late bariatric surgery complication occurring in 0.6–16% of Roux-en-Y gastric bypass (RYGB) patients [1, 2]. Comparable incidence was reported after one-anastomosis gastric bypass (OAGB), with a single study reporting a diagnosis of marginal ulcer in 9.5% of routine endoscopies 1 year post surgery [3, 4]. Identified risk factors for the development of marginal ulcers include nonsteroidal anti-inflammatory drug (NSAIDs), corticosteroid use and smoking, as well as foreign body reactions to staples or suture material, gastro-gastric fistulae and Helicobacter pylori infection [5]. Smoking and post-operative NSAIDs use were shown to be significant risk factors for recalcitrant ulcers necessitating revisional surgery [6]. Some concern exists relating to the constant bile flow over the anastomosis in OAGB anatomy [7]. However, it has not been shown to be as a clear risk factor for marginal ulcer development or response to treatment.

The majority of marginal ulcers heal with proton pump inhibitors (PPI) medical therapy, avoiding aggravating factors and *treating Helicobacter pylori* (HP) if diagnosed, with reported healing rates of up to 89% [8]. Dissolving the PPI was shown to further improve healing rates and time [9]. However, approximately half of the ulcers recur after PPI’s are stopped and others are refractory to medical treatment [10]. In cases of persistent ulcer patients eventually require surgical treatment. Surgical options include a redo of the gastro-jejunal anastomosis, omentopexy, conversion to sleeve gastrectomy, conversion to RYGB or a full reconstruction procedure. While a previous study described a success rate of up to 85% with surgical revision, another study reported ulcer recurrence in most patients [11, 12]. Furthermore, the choice of surgery greatly impacts the recurrence rates and is associated with morbidity [10].

Recently, a new technique of endoscopic suturing of a jejunal flap has been reported [13]. By covering the ulcer with jejunal tissue (flap) the ulcer is not exposed to acid, and this may promote healing. A self-expandable metal sent was inserted in cases of significant strictures that were caused by creating the flap or a pre-existing one. This approach was recently described for the first time in case series of 11 patients with refractory marginal ulcers post-Roux-en-Y gastric bypass with a success rate of 100% [13].

In this study, we aimed to evaluate the technical feasibility, clinical effectiveness and safety of the implementation of this approach in our practice.

## Methods

### Study design and patient population

Consecutive patients with marginal ulcers referred to Tel Aviv Medical Center between 2020 and 2024 were included. Initial endoscopy was performed to evaluate the size and location of the marginal ulcer, anastomotic diameter and rule out precipitating factors such as HP infection, gastro-gastric fistula and foreign material. Patients with a marginal ulcer of 10 mm or larger who were negative to HP and failed to respond to a two-month course of high-dose PPI (40 mg twice daily Esomeprazole or Pantoprazole) were defined as refractory and referred to endoscopic treatment. All patients were advised to avoid smoking, as well as NSAIDs and alcohol consumption. Patients were allowed to continue taking their regular medications including anti platelets agents. Exclusion criteria were the presence of a gastro-gastric fistula and an ulcer less than 10 mm in size or involving more than half of the anastomosis’ circumference.

### Endoscopic intervention

**Fig. 1:**
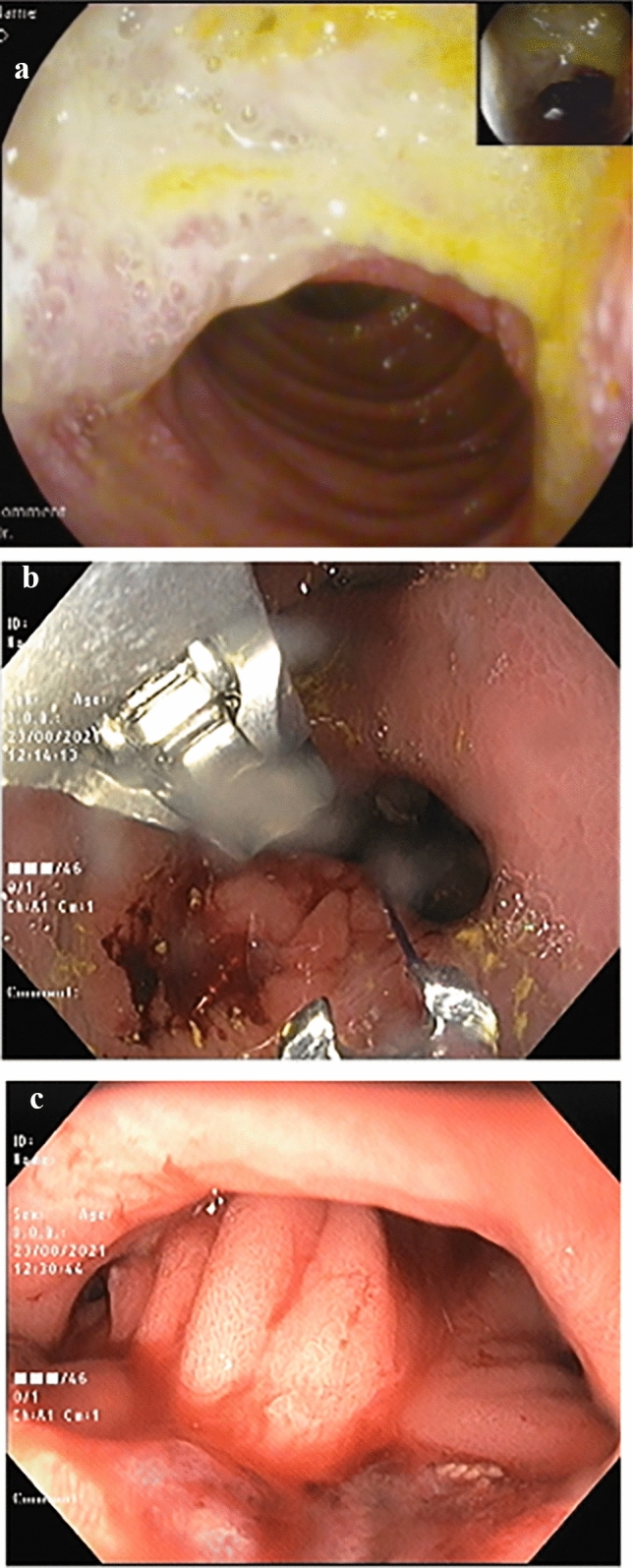
**a**–**c** demonstrates case number 4. **a** shows a marginal ulcer covering a third of the anastomosis, **b** shows the suturing of the jejunal flap and **c** shows the end result

**Fig. 2:**
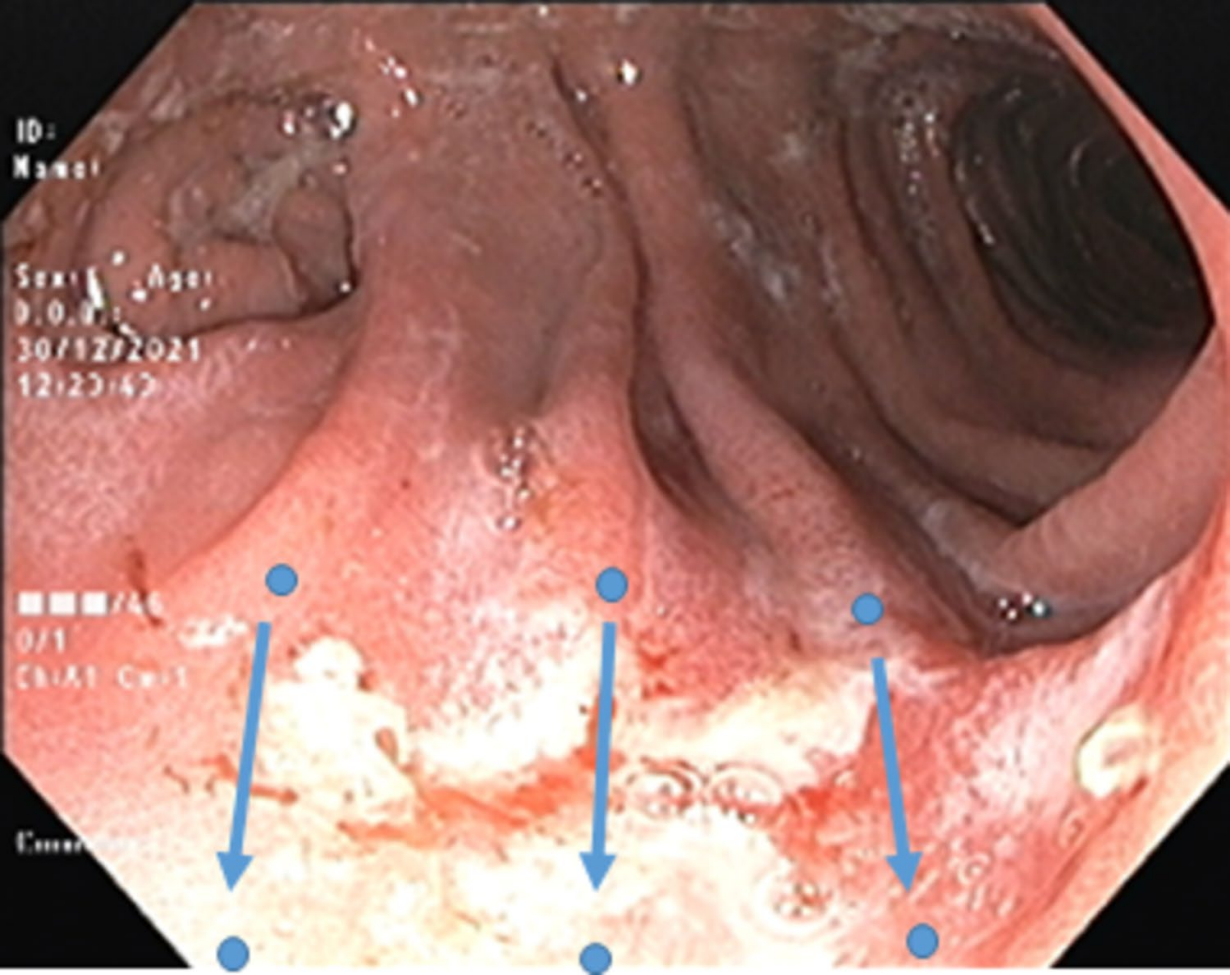
Illustrates the endoscopic technique of interrupted suture pattern. The dots represent each bite and the arrows the direction of suturing

All therapeutic endoscopies were performed under general anesthesia and endotracheal intubation, with the use of an esophageal overtube and carbon dioxide (CO2) insufflation. The jejunal flap was sutured using the Apollo Overstitch mounted on a double channel endoscope (Olympus EXERA II GIF-2 TH180 or Fujifilm ELUXEO EI-740D). Interrupted suturing was carried out until a jejunal flap fully covered the marginal ulcer (Fig. 1a–c). Each suture started with passing the needle in the jejunal side of the anastomosis proximal to the ulcer and a second pass was done in the gastric side, just proximal to the anastomosis Fig. 2). The suture was then cinched. The tissue helix was used for the gastric side to obtain full thickness bites. In cases where over-suturing the jejunal flap had resulted in an anastomotic diameter less than 10 mm, a stent was placed after suturing to prevent obstructive symptoms. In these cases, lumen apposing metal stents (Taewoong Spaxus 16 mm/20 mm) were used to reduce the chance of migration and avoid excessive radial forces on the ulcer. The stent was left in place for 8 weeks and then removed endoscopically.

### Outcomes

Technical success was defined as a marginal ulcer fully covered with a jejunal flap with or without stent inserted at the end of the procedure. Ulcer healing was evaluated endoscopically in all patients 8 weeks after suturing or stent removal. Clinical success was defined as a completely healed ulcer on endoscopy 8 weeks post procedure. Later follow up was done in the outpatient clinic or by phone call during which post procedural symptoms were recorded.

### Statistical analysis

Descriptive statistics were used to summarize patient demographics, clinical characteristics and outcome. Values for continuous variables were calculated and expressed as mean ± standard deviation. Frequencies and percentages were reported for categorical variables. All analyses and results were presented in tables below. Data were statistically analyzed using Excel (Microsoft, Redmond, WA, USA).

## Results

### Clinical characteristics of the cohort

Eleven consecutive patients, with marginal ulcers, were referred to our unit between 2020 and 2024 (Table 1). Seven (63.6%) were females, with a mean age of 53 ± 11.6 Seven (63.6%) patients had undergone RYGB and four (36.4%) OAGB. The mean BMI and time from surgery to marginal ulcer diagnosis were 24.1 kg/m2 ± 3.45 and 30.6 months ± 35, respectively. Four (36.4%) patients were active smokers at the time of diagnosis and another patient had a history of smoking. Two (18%) patients were on anti-thrombotic mediations, 1 aspirin and 1 clopidogrel and no patients were using anticoagulation therapy. Three (27.3%) patients had major comorbidities related to delayed wound healing: one had diabetes mellitus, 1 had hypertension and ischemic heart disease and 1 had diabetes mellitus, hypertension and ischemic heart disease. All patients were HP Negative confirmed by biopsy, denied alcohol abuse or NSAIDs use. All patients were treated with PPI at the time of diagnosis with average duration of 25.4 ± 34.5 months.

**Table 1:** Study population characteristics

# Patient	Age/Sex	BMI	Comorbidities	Smoking	PPI	PPI duration (months)	Antiplatelet/ anticoagulation therapy	Surgery type	Primary/ Revision surgery	Interval between Surgery and MU (months)
1	73/F	24	IHD, HTN, Diabetes	No	Capsule	132	Clopidogrel	RYGB	Primary	97
2	48/F	23.6	No	No	Soluble	24	No	RYGB	Primary	43
3	56/F	33.1	Diabetes	No	Capsule	24	No	OAGB	Revision	25
4	55/F	26.4	No	No	Soluble	6	No	RYGB	Revision	7
5	63/F	21.8	COPD, IHD, HTN	Past smoker	Soluble	11	Aspirin	OAGB	Primary	8
6	42/F	22.2	No	Active smoker	Capsule	24	No	RYGB	Primary	105
7	36/F	22.5	No	No	Capsule	10	No	OAGB	Primary	1
8	58/M	26	No	No	Soluble	6	No	RYGB	Revision	17
9	46/M	23	No	Active smoker	Capsule	12	No	RYGB	Revision	6
10	28/M	18.8	No	Active smoker	Capsule	7	No	OAGB	Revision	22
11	29/M2	23.6	No	Active smoker	Capsule	24	No	RYGB	Revision	6

*RYGB* Roux−en−Y gastric bypass, *OAGB* One−Anastomosis gastric bypass, *MU* Marginal ulcer, *BMI* Body mass index (calculated as body mass (Kg)/(body height)^2^, *PPI* Proton pump inhibitor, *IHD* Ischemic heart disease, *HTN* Hypertension, *COPD* Chronic obstructive pulmonary disease

### Technical and clinical effectiveness and safety

Technical success was achieved in 10 (90.1%) patients, with a jejunal flap sutured to the gastric pouch and fully covering the ulcer bed. In 2 (18%) of these patients a stent was inserted because of a narrowed anastomosis after the suturing (Table 2). In one patient suturing was not attempted as the anastomosis was too narrow to allow for the overstitch to pass through. The patient was treated with a stent only strategy to relieve the obstructive symptoms while awaiting surgery. Average number of sutures used was 2.8 ± 1.6 Previous sutures and/or staples were removed in 4 cases in the index endoscopy. No gastro-gastric fistulas were identified. All patients were hospitalized overnight and discharged the following day.

**Table 2:** Procedure data and endoscopic results

#Patient	Sutures number	Stent placement	Foreign body removal	Technical Success	Clinical success	Revision Surgery
1	2	No	Yes	Yes	Yes	No
2	2	No	No	Yes	Yes	No
3	7	No	No	Yes	No	OAGB- > RYGB
4	2	No	No	Yes	Yes	No
5	2	Yes	Yes	Yes	No	No
6	2	Yes	No	Yes	Yes	Reconstruction
7	0	Yes	Yes	No	No	Omentopexy
8	2	No	No	Yes	Yes	No
9	5	Yes	Yes	Yes	No	Re-anastomosis
10	2	No	No	Yes	No	No
11	2	No	No	Yes	No	Gastrectomy esophago-jejunal anastomosis

*RYGB* Roux−en−Y gastric bypass, *OAGB* One−anastomosis gastric bypass

**Fig. 3:**
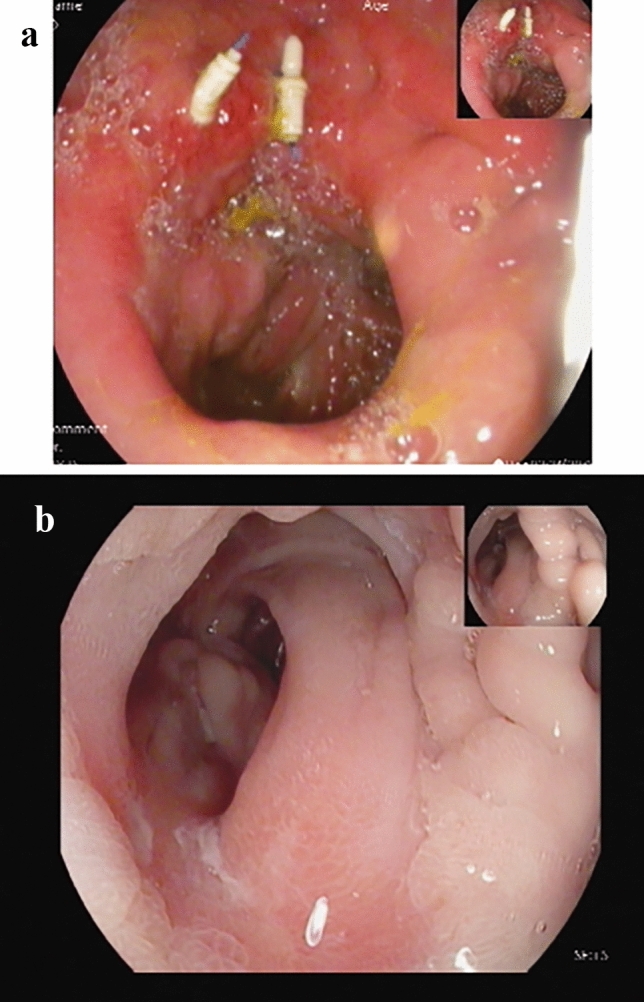
**a**–**b** demonstrates ulcer healing in follow-up endoscopy. **a** shows an intact jejunal flap, **b** shows complete resolution without evidence of the flap

In contrast to the high technical success rate, in only five (45.5%) patients complete ulcer healing was achieved at eight weeks follow up. An intact jejunal flap and sutures were noticed in 2 patients, while in the other 3 only ulcer healing was seen (Fig. 3a-b). Interestingly, all patients post OAGB experienced treatment failure, while 5 out of 7 patients following RYGB experienced clinical success (Table 3). Not surprisingly in two out of the three active smokers the endoscopic treatment failed. The fourth active smoker was treated only with a LAMS, and the ulcer had not healed as well. The patients in the clinical success group were generally older- 55 ± 10.5 vs 40 ± 19.8 years, with a longer time between the surgery and ulcer diagnosis- 53.8 ± 40.3 vs 13.4 ± 8.3 months and a longer exposure to PPI- 38.4 ± 47 vs 15.6 ± 7 months.

**Table 3:** Patients characteristics for clinical success/failure after jejunal flap suturing

	Clinical success (5)	Clinical failure (5)
Age mean (SD), years	55 (11.7)	44 (14)
BMI mean (SD)	24.4 (1.5)	24 (4.8)
RYGB/OAGB N	5 RYGB	4 OAGB, 1 RYGB
Interval between surgery and MU mean (SD), months	53.8 (40)	13.4 (8.3)
Number of patients with comorbidities	1	2
PPI duration mean (SD), months	38.4 (47)	15.6 (7)
Smoking N	1	2
Antiplatelets N	1 clopidogrel	1 aspirin

*BMI* Body mass index, *RYGB* Roux−en−Y gastric bypass, *OAGB* One−anastomosis gastric bypass, *MU* Marginal ulcer, *PPI* Proton pump inhibitors

During follow up of 7.6 months, five (45.5%) patients underwent surgical revision. One of which was not due to a non-healing marginal ulcer (patient number 6) but rather due to the continuation of abdominal pain even after ulcer resolution. She underwent a full reconstruction to normal anatomy. Unfortunately, her symptoms persisted even after this surgery. Different surgeries were performed in each of the other four patients. One patient had been converted from OAGB to RYGB with a redo of the anastomosis, one had an anastomosis redo, one had an urgent operation with omentopexy due to ulcer perforation and one had a gastrectomy with esophago-jejunal anastomosis.

No adverse events were observed during the endoscopic procedure or follow-up duration.

## Discussion

In this study we have shown the safety and effectiveness of endoscopic flap suturing in the treatment of refractory marginal ulcers following RYGB\OAGB. While the procedure was universally safe, its effectiveness in our experience was less than previously reported [13]. Overall technical success rate was 90.1%, but clinical success was seen only in 45% of the treated patients. One technical failure occurred, due to a constricted and edematous anastomosis not allowing the safe passage of the suturing device. Only a stent was used in this case to relief dysphagia symptoms and potentially expedite ulcer healing by covering it. The symptoms improved but the ulcer did not heal.

Marginal ulcers remain a dreaded late adverse event in patients undergoing bariatric surgery with incidence varies between 0.6 and 16%[1, 2], necessitating prolonged PPI use, repeat endoscopies and even revisional surgery. Several risk factors are associated with this adverse event, including smoking, alcohol use, NSAIDs and foreign material such as staples or sutures. Using endoscopic suturing may raise the concern of disturbing the ulcer healing by adding more sutures. However, a previous report by Kumbhari et al. [13] demonstrated a universal success rate using this approach for patients failing to response to high-dose PPI. Interestingly, smoking was found to be a risk factor for a recalcitrant ulcer in both series.

In our series a lower clinical success rate was achieved compared to the previous series. This may be attributed to a variance in the suturing technique used, or to a difference in the population. Indeed, several differences exist between the patient populations. First, our cohort was highly resistant to PPI as the average duration of PPI use was more than two years, these data were not disclosed in the previous series mentioned above. Second, there was a significant difference in the type of bypass surgery performed. OAGB has gained popularity in Israel and it is currently the leading bariatric surgery technique. Indeed, 4 of the 11 patients in this series underwent OAGB. All patients with marginal ulcers following OAGB experienced clinical failure, while 5 out of 7 patients following RYGB experienced clinical success. Of the two patients with clinical failure after RYGB one was an active smoker and the second was treated with a stent only strategy and did not undergo flap suturing due to anastomotic stricture. In comparison, in the previous series all cases were after RYGB.

Possible explanations to this observation may be the larger pouch typically seen after OAGB and\or the bile flow over the anastomosis that only occurs after OAGB, making it more prone to refractory marginal ulcers. Indeed, the average duration between surgery and diagnosis of marginal ulcer was 45.8 months after RYGB compared with 14 months after OAGB. Of note, previous reports did not show an increase in the incidence of marginal ulcer after OAGB [7].

Recent technological advancement has successfully enabled real-time visualization of tissue oxygenation using specialized endoscopic equipment (ELUXEO Vision, Fujifilm, Japan). This technology offers the potential to evaluate tissue perfusion and reduce the risk of anastomotic leakage [14]. It may also help identify ischemia as the etiology of a marginal ulcer and guide the decision on a therapeutic approach. This technology is not yet available in our region and could not be evaluated in these patients.

No adverse events (delayed bleeding or perforation) were observed during the procedure or in the follow-up period. No delayed stricture was observed, probably due to the use of a stent in the borderline cases. No stent migration occurred, demonstrating the advantages of using LAMS over standard SEMS in short strictures.

Several limitations of this study may also explain our results. This is a relatively small case series, and a larger series is needed to re-evaluate and confirm our findings. In addition, only half of the patients used soluble PPI but there was no correlation to clinical success compared to previous studies.

In conclusion, endoscopic suturing of a jejunal flap has a moderate success rate and was limited to patients post RYGB rather than post OAGB. Its excellent safety profile makes it a reasonable approach before referring to surgery. Further research is needed in order to assess its effectiveness in larger cohorts, define the patient population most likely to benefit from this intervention and determine a treatment algorithm.

## References

[1] Garrido AB Jr, Rossi M, Lima SE Jr, Brenner AS, Gomes CA Jr (2010) Early marginal ulcer following Roux-en-Y gastric bypass under proton pump inhibitor treatment: prospective multicentric study. Arq Gastroenterol 47(2):130–134 20721455 10.1590/s0004-28032010000200003

[2] Sapala JA, Wood MH, Sapala MA, Flake TM Jr (1998) Marginal ulcer after gastric bypass: a prospective 3-year study of 173 patients. Obes Surg 8(5):505–516 9819081 10.1381/096089298765554061

[3] Baksi A, Kamtam DNH, Aggarwal S, Ahuja V, Kashyap L, Shende DR (2020) Should surveillance endoscopy be routine after one anastomosis gastric bypass to detect marginal ulcers: initial outcomes in a tertiary referral centre. Obes Surg 30(12):4974–4980 32720263 10.1007/s11695-020-04864-y

[4] Magouliotis DE, Tasiopoulou VS, Tzovaras G (2019) One anastomosis gastric bypass versus Roux-en-Y Gastric Bypass for morbid obesity: an updated meta-analysis. Obes Surg 29(9):2721–2730 31172454 10.1007/s11695-019-04005-0

[5] Coblijn UK, Lagarde SM, de Castro SM, Kuiken SD, van Wagensveld BA (2015) Symptomatic marginal ulcer disease after Roux-en-Y gastric bypass: incidence, risk factors and management. Obes Surg 25(5):805–811 25381115 10.1007/s11695-014-1482-9

[6] Di Palma A, Liu B, Maeda A, Anvari M, Jackson T, Okrainec A (2021) Marginal ulceration following Roux-en-Y gastric bypass: risk factors for ulcer development, recurrence and need for revisional surgery. Surg Endosc 35(5):2347–2353 32424625 10.1007/s00464-020-07650-0

[7] Kupietzky A, Dodi O, Cohen N, Dover R, Maden A, Mazeh H, Grinbaum R, Mizrahi I (2024) Similar rates of symptomatic marginal ulcers after one-anastomosis-gastric bypass compared to Roux-en-Y Gastric Bypass. Obes Surg 34(7):2331–2337 38789681 10.1007/s11695-024-07298-y

[8] Azagury DE, Abu Dayyeh BK, Greenwalt IT, Thompson CC (2011) Marginal ulceration after Roux-en-Y gastric bypass surgery: characteristics, risk factors, treatment, and outcomes. Endoscopy 43(11):950–954 21997722 10.1055/s-0030-1256951

[9] Schulman AR, Chan WW, Devery A, Ryan MB, Thompson CC (2017) Opened proton pump inhibitor capsules reduce time to healing compared with intact capsules for marginal ulceration following Roux-en-Y gastric bypass. Clin Gastroenterol Hepatol 15(4):494–500.e1 27773764 10.1016/j.cgh.2016.10.015

[10] Bacoeur-Ouzillou O, Perinel J, Pelascini E, Abdallah M, Poncet G, Pasquer A, Robert M (2022) Management strategies of anastomotic ulcer after gastric bypass and risk factors of recurrence. Surg Endosc 36(12):9129–9135 35764841 10.1007/s00464-022-09393-6

[11] Patel RA, Brolin RE, Gandhi A (2009) Revisional operations for marginal ulcer after Roux-en-Y gastric bypass. Surg Obesity Related Dis 5(3):317–322 10.1016/j.soard.2008.10.011 19136312

[12] Moon RC, Teixeira AF, Goldbach M, Jawad MA (2014) Management and treatment outcomes of marginal ulcers after Roux-en-Y gastric bypass at a single high volume bariatric center. Surg Obesity Related Dis 10(2):229–234 10.1016/j.soard.2013.10.002 24462313

[13] Barola S, Fayad L, Hill C, Magnuson T, Schweitzer M, Singh V, Chen YI, Ngamruengphong S, Khashab MA, Kalloo AN, Kumbhari V (2018) Endoscopic management of recalcitrant marginal ulcers by covering the ulcer bed. Obes Surg 28(8):2252–2260 29556889 10.1007/s11695-018-3162-7

[14] Alomari M, Wadiwala I, Bowers S, Elli EF, Thomas M (2024) Oxygen saturation endoscopic imaging as a novel alternative to assess tissue perfusion during esophagectomy. Surg Innov 31(6):622–626 39361295 10.1177/15533506241290071

